# Supplementary material for: Sorting at embryonic boundaries requires high heterotypic interfacial tension
Source: Nat Commun. 2017 Jul 31;8:157. doi: 10.1038/s41467-017-00146-x (PMC5537356; doi:10.1038/s41467-017-00146-x)
Supplement: Supplementary file 2 — Supplementary Software 1 [file 41467_2017_146_MOESM2_ESM.zip › PottsModel/SrcPottsModel/doc/index-files/index-20.html]

U-Index


JavaScript is disabled on your browser.


Skip navigation links


- Overview
- Package
- Class
- Use
- Tree
- Deprecated
- Index
- Help

- Prev Letter
- Next Letter

- Frames
- No Frames

- All Classes

A B C D E F G H I J K L M N O P R S T U V W 


## U

update(Notification) - Method in class engine.PottsLogger


update(Notification) - Method in class engine.StatisticsManager


update(Notification) - Method in class gui.PlotPanel


update(Notification) - Method in class gui.PottsCanvas


update(Notification) - Method in class gui.PottsFrame


update(Notification) - Method in class gui.SnapshotManager


update(Notification) - Method in class gui.StatusBar


update(Notification) - Method in class model.Lattice


update(Notification) - Method in interface mvc.Observer
:   Determines what an observer should do upon notification that the observed object has changed.

updateAllPixelParameters() - Method in class model.Lattice


updateParameters(Pixel, boolean) - Method in class model.Cell
:   Call after a pixel has been added or removed from a cell

updateStatisticsManager(StatisticsManager) - Method in class gui.PottsFrame


utils - package utils


Utils() - Constructor for class engine.Statistic.Utils


Utils - Class in engine


Utils() - Constructor for class engine.Utils


Utils - Class in gui


Utils() - Constructor for class gui.Utils


Utils.EnergyTracker - Class in engine

A B C D E F G H I J K L M N O P R S T U V W

Skip navigation links


- Overview
- Package
- Class
- Use
- Tree
- Deprecated
- Index
- Help

- Prev Letter
- Next Letter

- Frames
- No Frames

- All Classes
